# Supplementary material for: HDAC9-mediated epithelial cell cycle arrest in G2/M contributes to kidney fibrosis in male mice
Source: Nat Commun. 2023 May 25;14:3007. doi: 10.1038/s41467-023-38771-4 (PMC10212923; doi:10.1038/s41467-023-38771-4)
Supplement: Supplementary file 1 — Supplementary Information [file 41467_2023_38771_MOESM1_ESM.pdf]

Supplementary materials

HDAC9-mediated epithelial cell cycle arrest in G2/M contributes to kidney fibrosis in male mice

Yang Zhang[1], Yujie Yang[1], Fan Yang[2], Xiaohan Liu[1], Ping Zhan[1], Jichao Wu[1], Xiaojie Wang[1], Ziying Wang[1], Wei Tang[1], Yu Sun[1], Yan Zhang[1], Qianqian Xu[3], Jin Shang[4], Junhui Zhen[5], Min Liu*[1], Fan Yi*[1]

[1]The Key Laboratory of Infection and Immunity of Shandong Province, Department of Pharmacology, School of Basic Medical Sciences, Shandong University, Jinan, 250012, China.
[2]Department of Neurosurgery, Provincial Hospital Affiliated to Shandong First Medical University, Jinan, 250021, China.
[3]Department of Organ Transplantation, Qilu Hospital of Shandong University, Jinan, 250012, China.
[4]Department of Nephrology, the First Affiliated Hospital of Zhengzhou University, Zhengzhou, 450052, China.
[5]Department of Pathology, School of Basic Medical Sciences, Shandong University, Jinan, 250012, China.

***Corresponding Author:**
Fan Yi, The Key Laboratory of Infection and Immunity of Shandong Province, Department of Pharmacology, School of Basic Medical Sciences, Shandong University, Jinan, 250012, China. Email: fanyi@sdu.edu.cn;

Min Liu, The Key Laboratory of Infection and Immunity of Shandong Province, Department of Pharmacology, School of Basic Medical Sciences, Shandong University, Jinan, 250012, China. Email: liuweimin@sdu.edu.cn.

Running Title: HDAC9 deficiency attenuated kidney fibrosis

Inventory of supplementary materials

I. Supplementary tables

Table S1. Clinical characteristics in the normal human control subjects or subjects with chronic kidney disease.
Table S2. Physical and biochemical parameters of mice with *HDAC9* deficiency.
Table S3. Physical and biochemical parameters of mice with *HDAC9* deficiency in tubules.
Table S4. Primer pairs of target genes used for PCR in this study.
Table S5. Antibodies used in this study.

II. Supplementary figures

Supplementary Figure S1. HDAC9 was significantly increased in tubular epithelial cells.
Supplementary Figure S2. Establishment of *HDAC9* knockout ($HDAC9^{-/-}$) mice.
Supplementary Figure S3. *HDAC9* deficiency attenuated kidney fibrosis.
Supplementary Figure S4. Establishment of tubule-specific *HDAC9* knockout ($Cre^{+}/HDAC9^{fl/fl}$) mice.
Supplementary Figure S5. *HDAC9* knockdown reduced the expression of markers associated with fibrosis and proinflammatory mediators in HK-2 with TGF-β1 treatment.
Supplementary Figure S6. *HDAC9* deficiency inhibited tubular epithelial cell G2/M arrest.
Supplementary Figure S7. HDAC9 contributed to epithelial cell cycle arrest in G2/M and activation of fibroblasts.
Supplementary Figure S8. *HDAC9* upregulated the expression of TGF-β1, CTGF and p21.
Supplementary Figure S9. STAT1 was involved in HDAC9-mediated fibrotic effect.
Supplementary Figure S10. Tubule-specific deletion of *HDAC9* reduced the expression of Vimentin and α-SMA.
Supplementary Figure S11. TMP195 attenuated kidney fibrosis and inhibited G2/M phase arrest in TECs.
Supplementary Figure S12. DNMT3a was increased in fibrotic kidneys and HK-2 with AA or TGF-β1 treatment.

Supplementary tables

Table S1. Clinical characteristics in the normal human control subjects or subjects with chronic kidney disease.

Normal group (n = 6)	
Age (years)	50.17±4.97
Gender (male, n, %)	2 (33.33%)
SCr (μmol/l)	64.33±4.72
BUN (mmol/l)	4.92±0.56
eGFR (ml/min/1.73 m^2)	75.14±4.28
Subjects with renal fibrosis (n = 17)	
Age (years)	50.47±2.72
Gender (male, n, %)	13 (76.47%)
SCr (μmol/l)	158.06±10.15 ($p < 0.0001$)[#]
BUN (mmol/l)	11.50±1.78 ($p = 0.0214$)*
eGFR (ml/min/1.73 m^2)	33.45±2.23 ($p < 0.0001$)*
Pathological diagnosis	N (%)
Focal segmental glomerulosclerosis	9 (52.94%)
IgA nephropathy	5 (29.41%)
Diabetic nephropathy	3 (17.65%)

SCr, serum creatinine; BUN, blood urea nitrogen; eGFR, estimated glomerular filtration rate; # Mann-Whitney rank sum test with two-tailed *p*-value; * Unpaired t-test with two-tailed *p*-value; SEM, Standard error of mean; Data are expressed as mean ± SEM.

Table S2. Physical and biochemical parameters of mice with *HDAC9* deficiency.

Variables		$HDAC9^{+/+}$	$HDAC9^{-/-}$
Body weight (g)		26.25 ±0.94	26.37±0.59
kidney weight (g)		0.26±0.01	0.26±0.02
Heart rate (beat/min)		490.56±14.40	496.89±17.36
Blood pressure (mmHg)	Systolic	112.11±2.98	110.84±2.77
	Diastolic	72.28±3.13	71.45±2.75
Scr (μmol/l)		9.65±0.64	9.08±0.73
BUN (mmol/l)		8.74±0.41	8.98±0.49

n = 6 mice per group. Data are expressed as mean ± SEM

Table S3. Physical and biochemical parameters of mice with *HDAC9* deficiency in tubules.

Variables		$Cre^{-}/HDAC9^{fl/fl}$	$Cre^{+}/HDAC9^{fl/fl}$
Body weight (g)		26.05 ±0.76	26.22±0.81
kidney weight (g)		0.25±0.02	0.26±0.01
Heart rate (beat/min)		487.00±20.08	490.78±17.48
Blood pressure (mmHg)	Systolic	111.39±4.29	112.44±2.86
	Diastolic	72.00±2.99	72.39±2.23
Scr (μmol/l)		9.32±0.52	8.95±0.67
BUN (mmol/l)		9.01±0.51	8.83±0.73

n = 6 mice per group. Data are expressed as mean ± SEM

Table S4. Primer pairs of target genes used for PCR in this study.

Gene	Species	Forward (5' to 3')	Reverse (5' to 3')
Global *HDAC9* knockout	Mouse	P1 CCCCAAATTACCTCGCACCTTA	P2 CCTCCCGGATTCCACTTGTT
		P3 CGTGGGCCGTCTCTGTCTTG	P4 CCCTGGTGCCTCTTACTTTCTCG
$HDAC9^{fl/fl}$	Mouse	AAGCAGCACGAGAATTTGAC	CTAAGGAAGAACTACATTGCCCTAT
Cdh16-Cre	Mouse	P1 GCAGATCTGGCTCTCCAAAG	P2 AGGCAAATTTTGGTGTACGG
		P3 CAAATGTTGCTTGTCTGGTG	P4 GTCAGTCGAGTGCACAGTTT
HDAC9	Mouse	AGTGCGAGACACAGATGCTC	TACATGAACGTGGCTGGAGG
p21	Mouse	TTGTCGCTGTCTTGCACTCT	TAGAAATCTGTCAGGCTGGTCT
β-actin	Mouse	GGCTGTATTCCCCTCCATCG	CCAGTTGGTAACAATGCCATGT
HDAC9	Human	CTCATGCCGAGCAAATGGTT	GGAACACCTTGCCTAAGCGT
CTGF	Human	TTAGCGTGCTCACTGACCTG	GCCACAAGCTGTCCAGTCTA
β-actin	Human	CATGTACGTTGCTATCCAGGC	CTCCTTAATGTCACGCACGAT
α-SMA	Rat	CCTCATGCCATCATGCGTCT	CTCACGCTCAGCAGTAGTCA
β-actin	Rat	CTCTGTGTGGATTGGTGGCT	CGCAGCTCAGTAACAGTCCG
IL-6	Mouse	CTTCTTGGGACTGATGCTGGT	CTCTGTGAAGTCTCCTCTCCG
TNFα	Mouse	CAGGCGGTGCCTATGTCTC	CGATCACCCCGAAGTTCAGTAG
IL-1β	Mouse	CTGCAGCTGGAGAGTGTGG	GGGGAACTCTGCAGACTCAA
IL-1β	Human	ATGATGGCTTATTACAGTGGCAA	GTCGGAGATTCGTAGCTGGA
IL-6	Human	ACTCACCTCTTCAGAACGAATTG	CCATCTTTGGAAGGTTCAGGTTG
TNFa	Human	CCTCTCTCTAATCAGCCCTCTG	GAGGACCTGGGAGTAGATGAG

Table S5. Antibodies used in this study.

Primary antibodies	Source	Provider	Catalog	Application
HDAC9	Rabbit	ORIGENE	TA324378	WB (1:500), IHC (1:50), IF (1:50)
Fibronectin	Rabbit	ProteinTech Group	15613-1-AP	WB (1:1000)
Vimentin	Rabbit	Abcam	ab92547	IHC (1:100), IF (1:100)
Vimentin	Rabbit	Cell Signaling Technology	5741	WB (1:1000)
Alpha-smooth muscle	Rabbit	Abcam	ab124964	WB (1:2000), IHC (1:100), IF (1:100)
PCNA	Rabbit	ProteinTech Group	10205-2-AP	WB (1:1000), IHC (1:50), IF (1:100)
Collagen I	Rabbit	Affinity	AF7001	WB (1:1000)
Collagen I	Rabbit	Cell Signaling Technology	72026	IHC (1:50), IF (1:50)
Collagen IV	Rabbit	Abcam	ab236640	IHC (1:50)
Cyclin B1	Mouse	Cell Signaling Technology	4135	IHC (1:50), IF (1:50)
Cyclin B1	Rabbit	ProteinTech Group	55004-1-AP	WB (1:1000)
Cyclin D1	Rabbit	Abcam	ab16663	WB (1:1000)
Ki-67	Rabbit	Abcam	ab15580	IF (1:50)
Histone H3 (phospho S10)	Mouse	Abcam	ab14955	IF (1:50)
PDGF Receptor β	Rabbit	Cell Signaling Technology	3169	IF (1:50)
Alpha-smooth muscle	Mouse	ProteinTech Group	67735-1-lg	IF (1:100)
TGF beta 1	Rabbit	Abcam	ab215715	IHC (1:50)
TGF beta 1	Rabbit	Abcam	ab179695	WB (1:2000)
STAT1 (phospho S727)	Rabbit	Abcam	ab109461	WB (1:1000), IHC (1:50)
Acetylated-Lysine Antibody	Rabbit	Cell Signaling Technology	9441	WB (1:1000)
STAT1	Mouse	Cell Signaling Technology	9176	IP (10μg), WB (1:1000)
STAT1	Rabbit	Abcam	ab234400	WB (1:1000)

STAT1	Mouse	Abcam	ab155933	IP (10μg), WB (1:1000)
p21	Rabbit	Abcam	ab109199	WB (1:1000), IF (1:50)
STAT1 (phospho S727)	Rabbit	Cell Signaling Technology	9177	IF (1:50)
HDAC3	Rabbit	ABclonal	A2139	WB (1:1000)
HDAC7	Rabbit	Abcam	ab166911	WB (1:1000)
HDAC8	Rabbit	ABclonal	A5829	WB (1:1000)
F4/80	Rabbit	Cell Signaling Technology	70076	IHC (1:50)
DNMT3a	Rabbit	Cell Signaling Technology	3598	WB (1:1000)
STAT2	Rabbit	ABclonal	A3588	WB (1:1000)
STAT3	Rabbit	ABclonal	A1192	WB (1:1000)
STAT4	Rabbit	ABclonal	A4523	WB (1:1000)
STAT5	Rabbit	ABclonal	A5029	WB (1:1000)
STAT6	Rabbit	ABclonal	A19120	WB (1:1000)
STAT2 (phospho Y690)	Rabbit	Abcam	ab191601	WB (1:1000)
STAT3 (phospho Tyr705)	Rabbit	Cell Signaling Technology	9145	WB (1:1000)
STAT4 (phospho Y690)	Mouse	Santa Cruz Biotechnolog	sc-28296	WB (1:500)
STAT5 (phospho Tyr694)	Rabbit	Cell Signaling Technology	4322	WB (1:500)
STAT6 (phospho Y641)	Rabbit	ABclonal	AP0456	WB (1:500)
Mouse IgG1 Isotype Control	Mouse	Cell Signaling Technology	5415	IP (10μg)
Mouse Anti-rabbit IgG	Mouse	Cell Signaling Technology	5127	WB (1:2000)
Goat Anti-Rabbit IgG (H+L) HRP	Goat	Abways Technology	AB0101	WB (1:10000)
Goat Anti-Mouse IgG (H+L) HRP	Goat	Abways Technology	AB0102	WB (1:10000)
Goat Anti-Rabbit IgG H&L (Alexa Fluor® 594)	Goat	Abcam	ab150080	IF (1:200)
Goat Anti-Mouse IgG H&L (Alexa Fluor® 594)	Goat	Abcam	ab150116	IF (1:200)
Goat Anti-Rabbit IgG H&L (Alexa Fluor® 488)	Goat	Abcam	ab150077	IF (1:200)

GAPDH	Rabbit	Abways Technology	AB0037	WB (1:10000)
β-actin	Rabbit	Abways Technology	AB0035	WB (1:10000)
AQP1	Mouse	Abcam	ab9566	IF (1:100)
THP	Mouse	Santa Cruz Biotechnolog	sc-271022	IF (1:100)

Supplementary figures

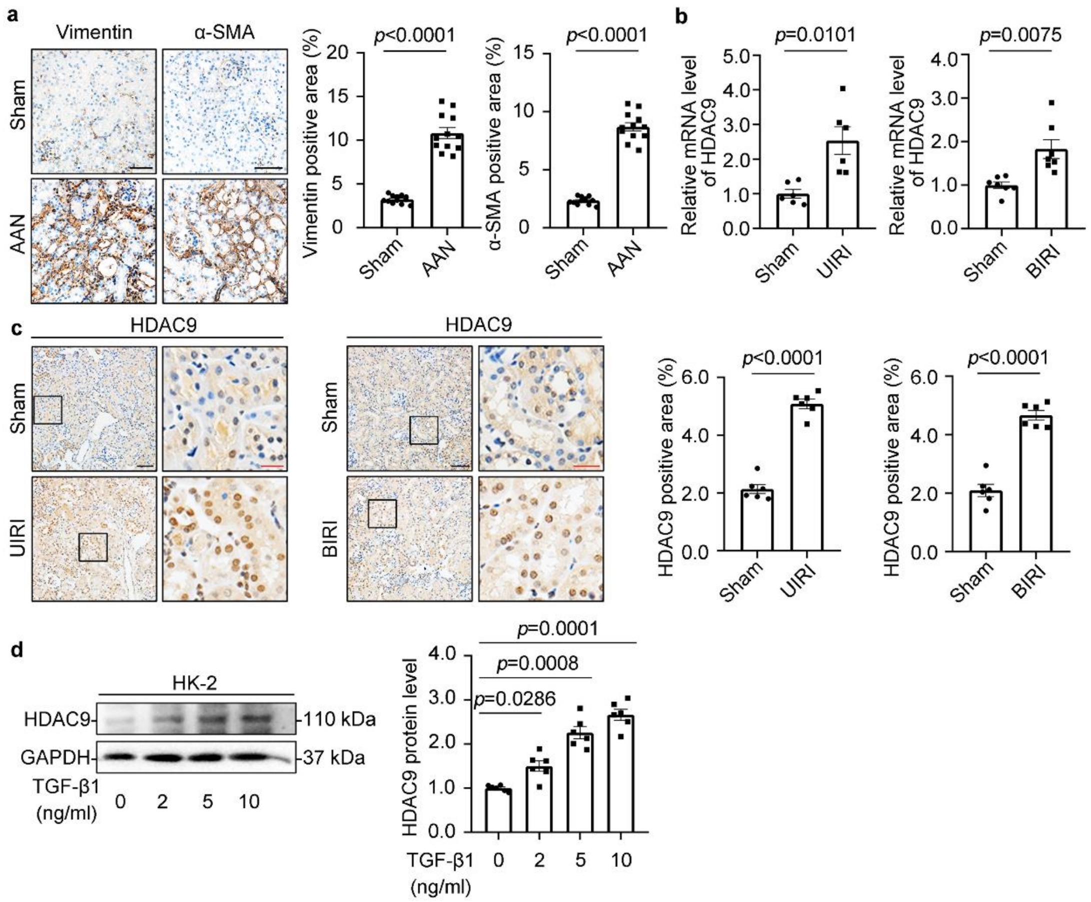

Supplementary Figure S1. HDAC9 was significantly increased in tubular epithelial cells. a. Photomicrographs and quantifications of Vimentin and α-SMA in kidney from AAN mice. Scale bar: black = 50 μm. (n = 12 mice per group). **b.** Relative mRNA level of *HDAC9* in the cortex of kidney from UIRI (n = 6 mice per group) and BIRI (n = 7 mice per group) mice. **c.** Photomicrographs and quantifications showing the expression of HDAC9 in the cortex of kidney from UIRI (n = 6 mice per group) and BIRI (n = 6 mice per group) mice. Bar: black = 50 μm; red = 20 μm. **d**. Representative Western blot gel documents and summarized data showing the relative protein levels of HDAC9 in human tubule epithelial cells (HK-2) with TGF-β1 treatment for 48 h. (n = 6 biologically independent experiments). Data are expressed as mean ± SEM (a-d). Two-tailed Student's unpaired t test analysis (a-c). One-way ANOVA followed by Tukey's post-test (d). Source data are provided as a Source Data file.

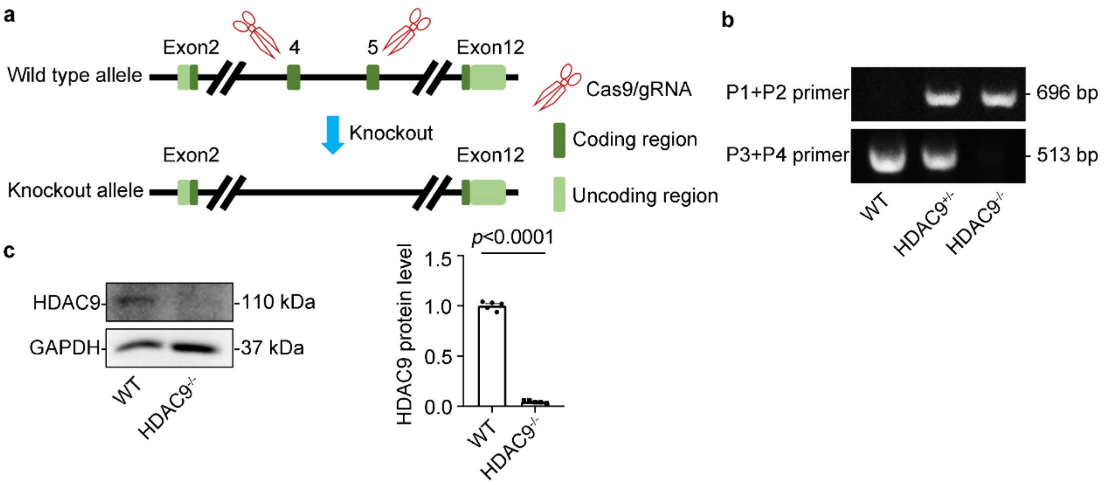

Supplementary Figure S2. Establishment of *HDAC9* knockout (*HDAC9*$^{-/-}$) mice. a. Scheme for *HDAC9* knockout mice. **b**. Genotyping was confirmed by tail preparation and PCR at 2 weeks of age. **b** was repeated five times independently with similar results. **c**. Representative Western blot gel documents and summarized data showing the relative protein levels of HDAC9 in the cortex of kidney from *HDAC9*$^{-/-}$ mice. (n = 5 mice per group). Data are expressed as mean ± SEM (c). Two-tailed Student's unpaired t test analysis (c). Source data are provided as a Source Data file.

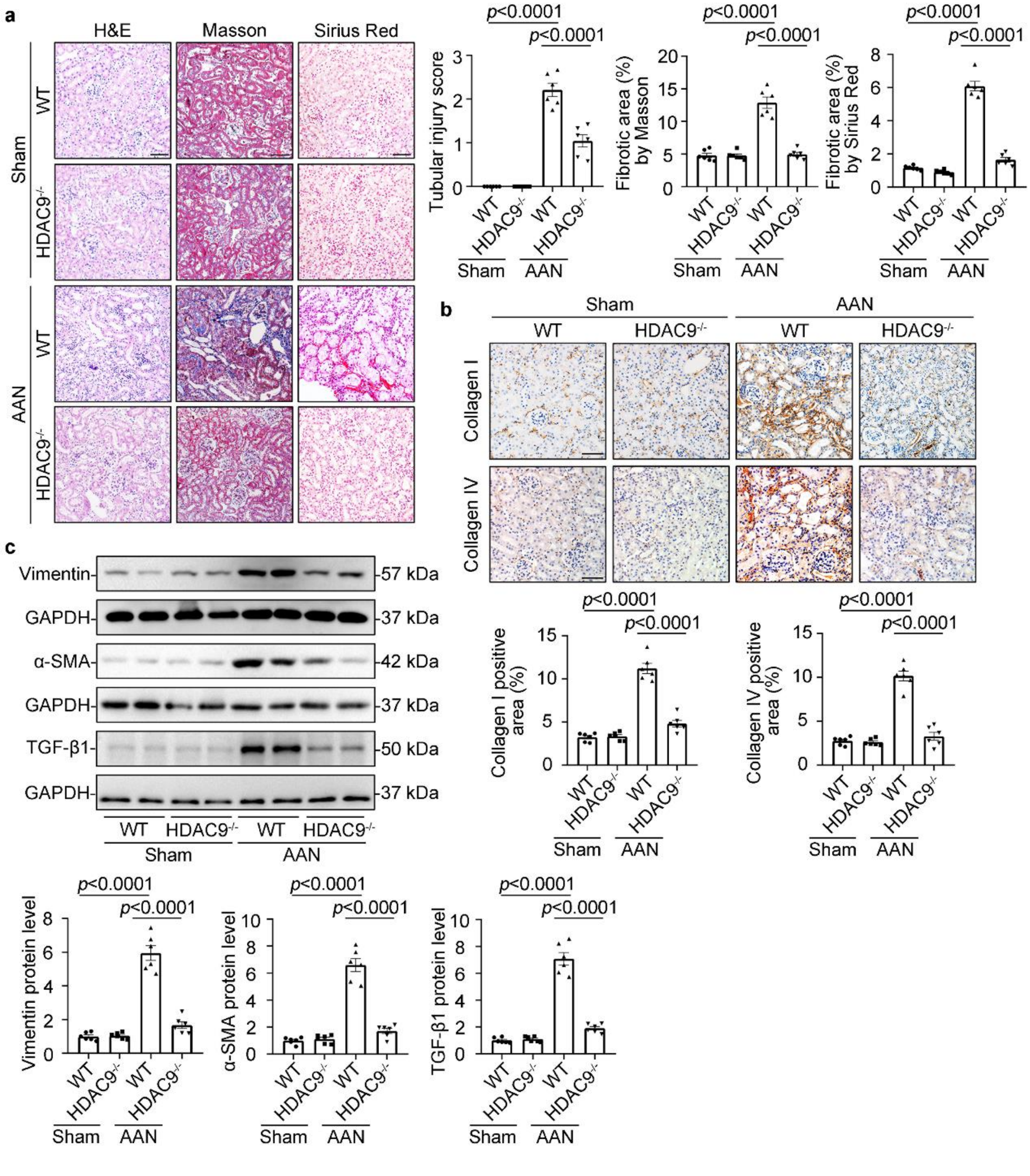

Supplementary Figure S3. ***HDAC9*** **deficiency attenuated kidney fibrosis. a.** H&E staining, Masson´s trichrome staining and Sirius Red staining were performed to assess the kidney injury and fibrosis in different groups. Scale bar: black = 50 μm. (n = 6 mice per group). **b.** Photomicrographs and quantifications showing the expression of collagen I and collagen IV in kidney from different groups of mice. Scale bar: black = 50 μm. (n = 6 mice per group). **c.** Representative Western blot gel documents and summarized data showing the relative protein levels of Vimentin, α-SMA and TGF-β1 in the cortex of kidney from different groups of mice. (n = 6 mice per group). Data are expressed as mean ± SEM (a-c). Two-way ANOVA followed by Tukey's post-test (a-c). Source data are provided as a Source Data file.

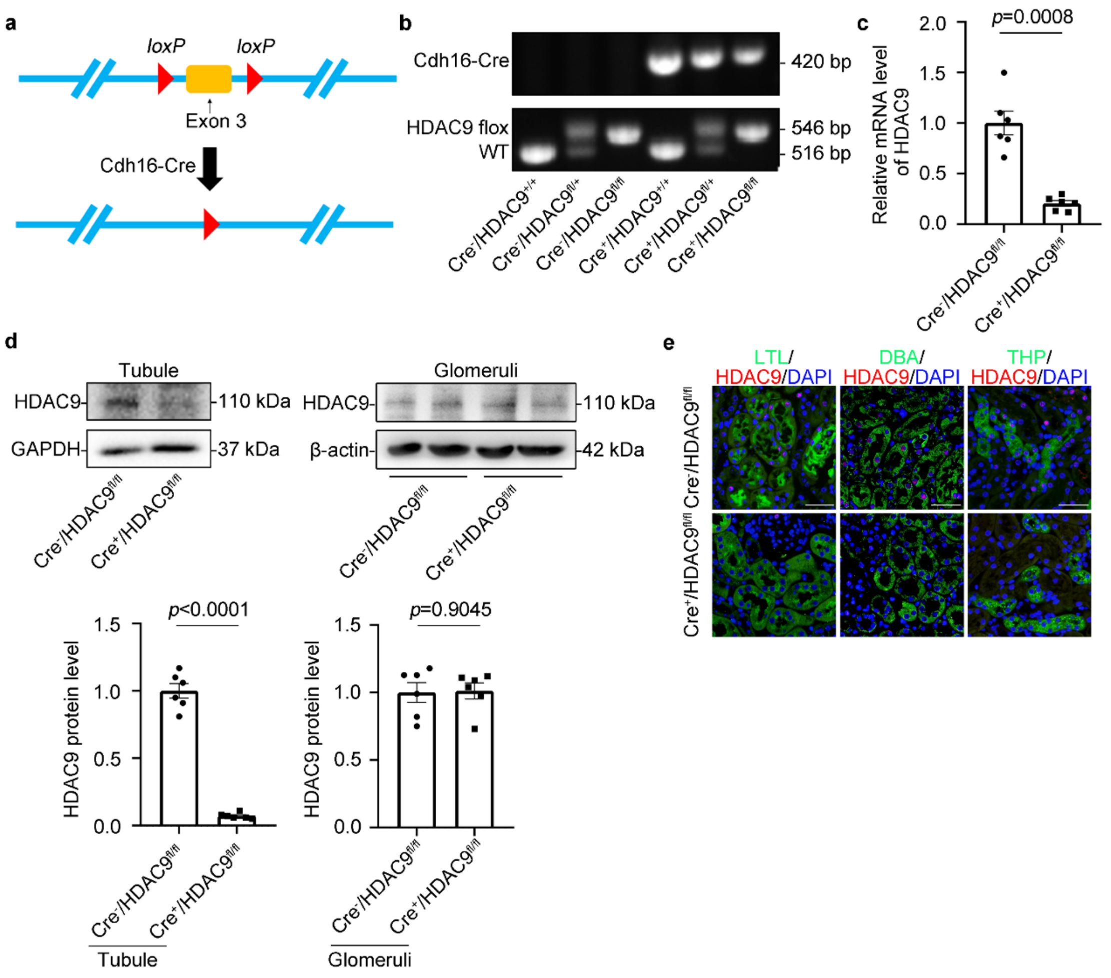

Supplementary Figure S4. Establishment of tubule-specific *HDAC9* knockout (*Cre*$^{+}$*/HDAC9*$^{fl/fl}$) mice. a. Generation of conditional knockout mice in which *HDAC9* is specifically ablated renal tubular cells by using *Cre–LoxP* recombination system. Exon 3 is deleted upon Cdh16-Cre–mediated recombination. **b**. Genotyping was confirmed by tail preparation and PCR at 2 weeks of age. This experiment was repeated five times independently with similar results. **c.** Relative mRNA level of *HDAC9* in the cortex of kidney from *Cre*$^{+}$*/HDAC9*$^{fl/fl}$ mice. (n = 6 mice per group) **d**. Representative Western blot gel documents and summarized data showing the relative protein levels of HDAC9 in isolated tubule or glomeruli from *Cre*$^{+}$*/HDAC9*$^{fl/fl}$ mice. (n = 6 mice per group). **e**. Photomicrographs showed that HDAC9 was deleted in renal tubules, including proximal tubule. Lotus tetragonolobus lectin (LTL) was used as a proximal tubular marker. Tamm-Horsfall glycoprotein (THP) was used as a marker for ascending loop of Henle; dolichos biflorus agglutinin (DBA) was used as a marker for collecting duct. Scale bar: white = 20 µm. This experiment was repeated three times independently with similar results. Data are expressed as mean ± SEM (c and d). Two-tailed Student's unpaired t test analysis (c-d). Source data are provided as a Source Data file.

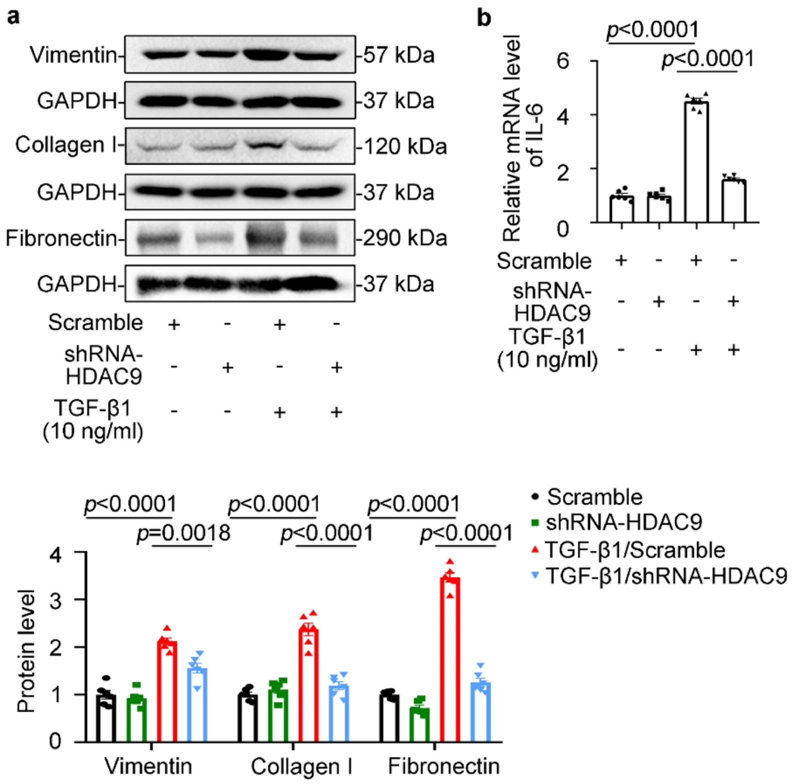

Supplementary Figure S5. ***HDAC9*** **knockdown reduced the expression of markers associated with fibrosis and proinflammatory mediators in HK-2 with TGF-β1 treatment. a.** Representative Western blot gel documents and summarized data showing the relative protein levels of Vimentin, Collagen I and Fibronectin in HK-2 with TGF-β1 treatments. (n = 6 biologically independent experiments). **b and c.** Relative mRNA level of *IL-6* and *TNFα* in HK-2 with different treatments. (n = 6 biologically independent experiments). Data are expressed as mean ± SEM (a-c). Two-way ANOVA followed by Tukey's post-test (a-c). Source data are provided as a Source Data file.

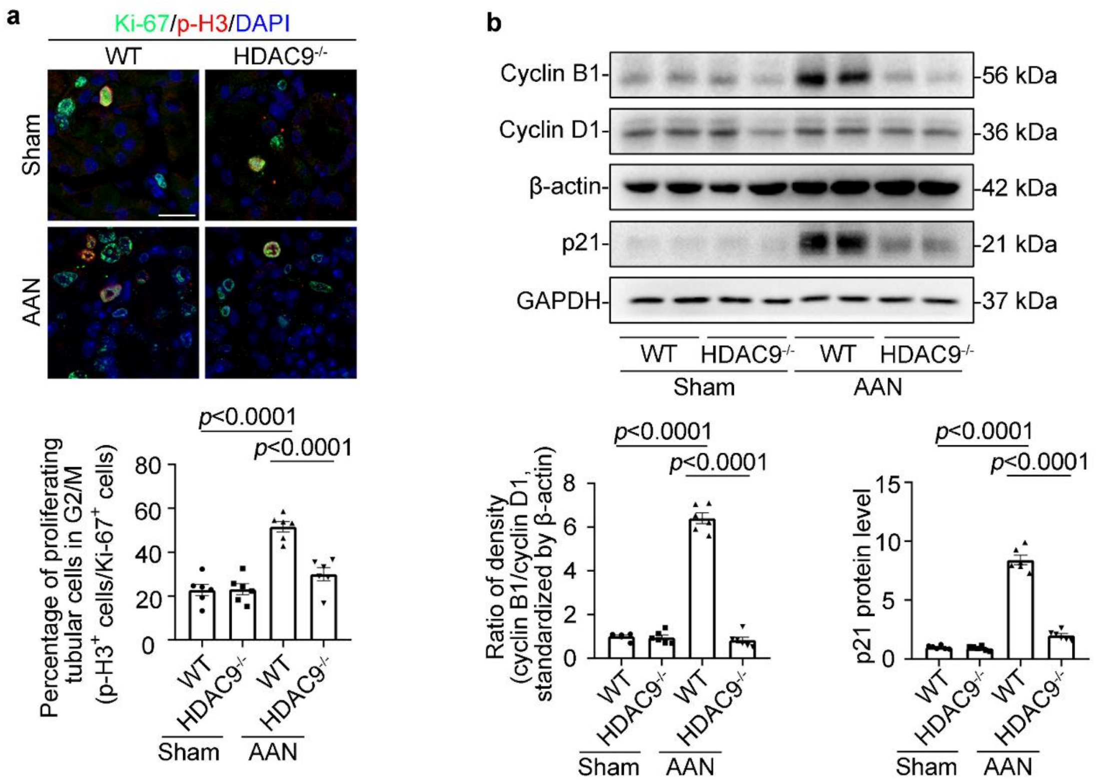

Supplementary Figure S6. ***HDAC9*** **deficiency inhibited tubular epithelial cell G2/M arrest. a.** Representative photomicrographs of coimmunostaining with antibodies to Ki-67 (anti–Ki-67) and p-H3 (anti–p-H3) on kidneys and the percentage of Ki-67^{+} p-H3^{+} cells among total Ki-67^{+} tubular cells in different groups. Scale bar: white = 10 μm. (n = 6 mice per group). **b.** Representative Western blot gel documents and summarized data showing the ratio of cyclin B1 to cyclin D1 densities standardized to β-actin and the relative protein levels of p21 in the cortex of kidney from different groups of mice. (n = 6 mice per group). Data are expressed as mean ± SEM (a and b). Two-way ANOVA followed by Tukey's post-test (a and b). Source data are provided as a Source Data file.

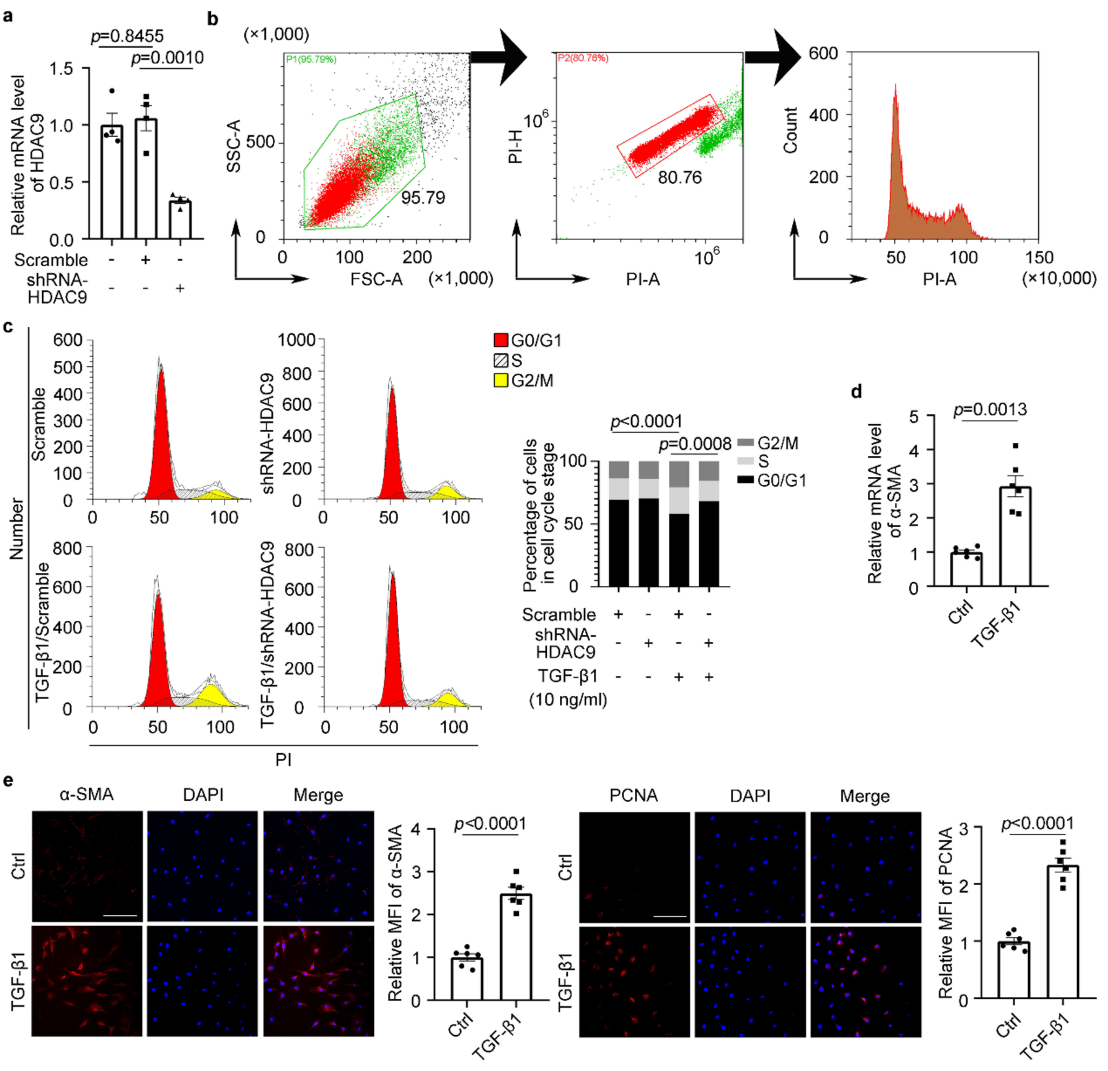

Supplementary Figure S7. HDAC9 contributed to epithelial cell cycle arrest in G2/M and activation of fibroblasts. a. The relative mRNA level of *HDAC9* in HK-2 with *HDAC9*-lentivirus transfection. (n = 4 biologically independent experiments). **b**. Representative gating strategy for analyzing the G2/M cell cycle arrest. **c.** Cell cycle analysis by flow cytometry for HK-2 in different groups. (n = 6 biologically independent experiments). Red: G0/G1; yellow: G2/M. **d**. Relative mRNA level of *α-SMA* in NRK-49F with different treatments. (n = 6 biologically independent experiments). **e.** Photomicrographs and quantifications showing the expression of α-SMA (red) and PCNA (red) in NRK-49F with TGF-β1 (5 ng/ml) treatment for 24h. Scale bar: white = 100 μm. (n = 6 biologically independent experiments). Data are expressed as mean ± SEM (a, c, d, e). Two-way ANOVA followed by Tukey's post-test (a and c). Two-tailed Student's unpaired t test analysis (d and e). Source data are provided as a Source Data file.

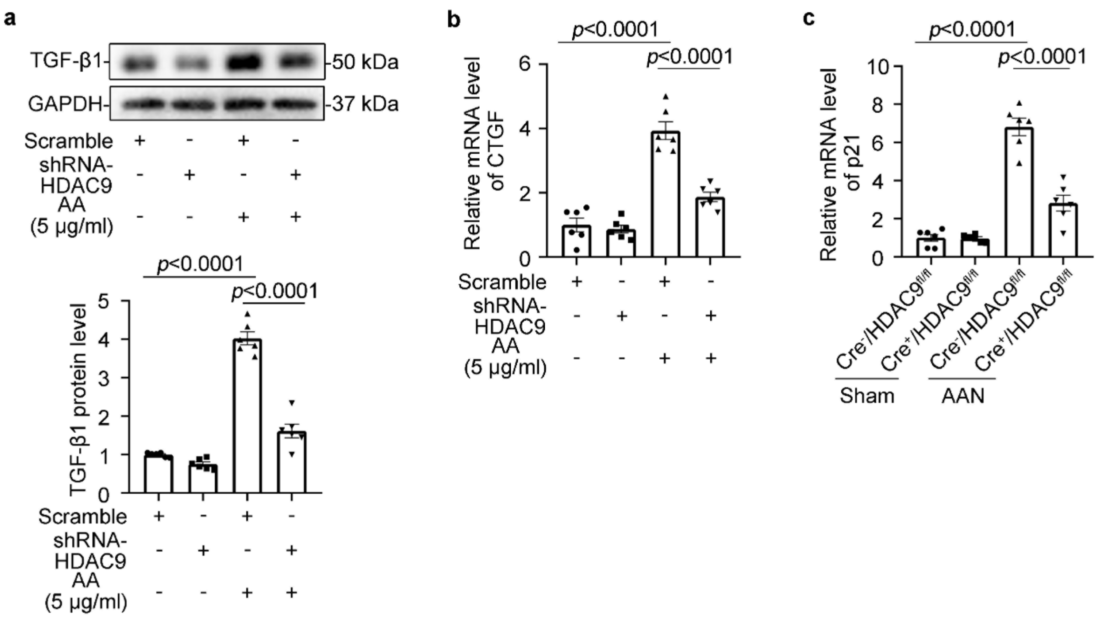

Supplementary Figure S8. ***HDAC9*** **upregulated the expression of TGF-β1, CTGF and p21. a.** Representative Western blot gel documents and summarized data showing the relative protein levels of TGF-β1 in HK-2 with AA treatment. (n = 6 biologically independent experiments). **b.** Relative mRNA level of *CTGF* in HK-2 with different treatments. (n = 6 biologically independent experiments). **c.** Relative mRNA level of *p21* in isolated tubules from different groups of mice. (n = 6 mice per group). Data are expressed as mean ± SEM (a-c). Two-way ANOVA followed by Tukey's post-test (a-c). Source data are provided as a Source Data file.

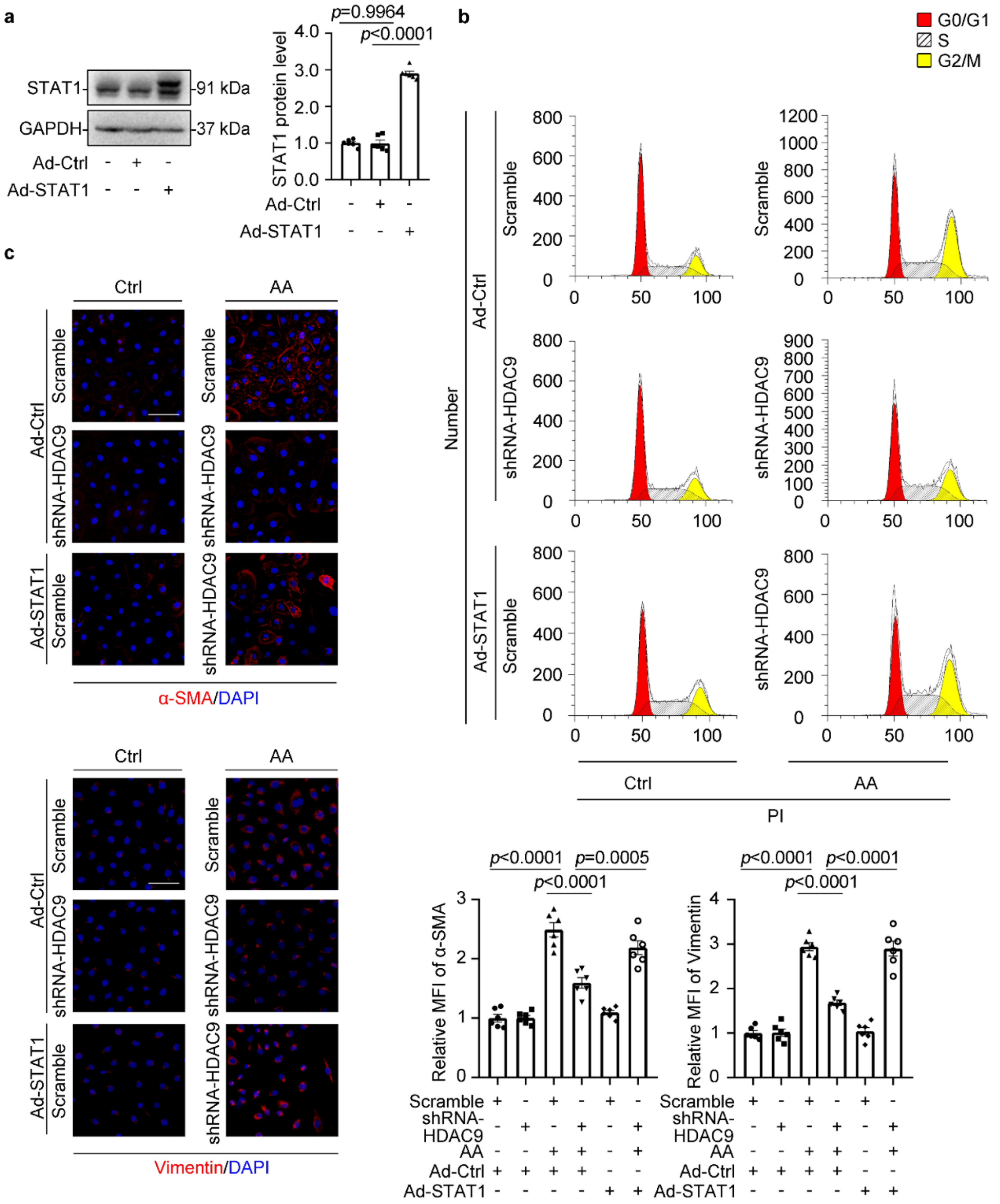

Supplementary Figure S9. STAT1 was involved in HDAC9-mediated fibrotic effect. a. Representative Western blot gel documents and summarized data showing the relative protein level of STAT1 in HK-2 with STAT1-adenovirus transfection. (n = 6 biologically independent experiments). **b**. Cell cycle analysis by flow cytometry for HK-2 in different groups. Red: G0/G1; yellow: G2/M. **c**. Photomicrographs and quantifications showing the relative protein levels of α-SMA, and Vimentin in HK-2 with different treatments. Scale bar: white = 50 μm. (n = 6 biologically independent experiments). Data are expressed as mean ± SEM (a and c). Two-way ANOVA followed by Tukey's post-test (a and c). Source data are provided as a Source Data file.

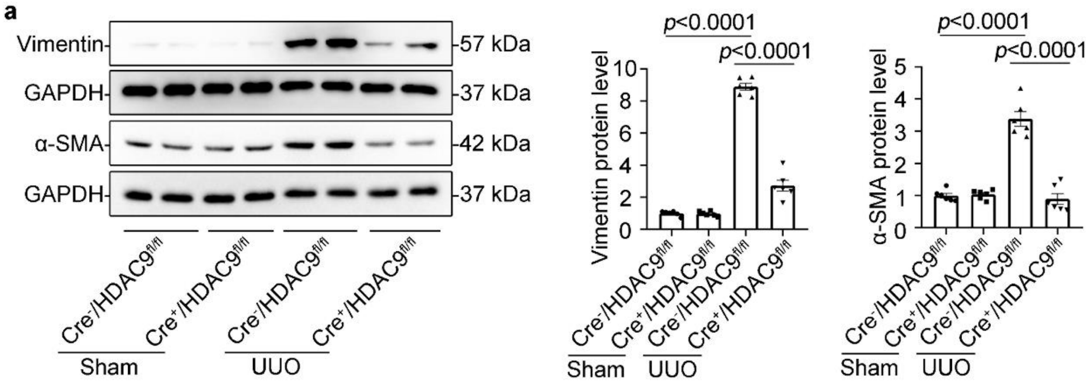

Supplementary Figure S10. Tubule-specific deletion of *HDAC9* reduced the expression of Vimentin and α-SMA. a. Representative Western blot gel documents and summarized data showing the relative protein levels of Vimentin and α-SMA in cortex of kidney from different groups of mice. (n = 6 mice per group). Data are expressed as mean ± SEM (a). Two-way ANOVA followed by Tukey's post-test (a). Source data are provided as a Source Data file.

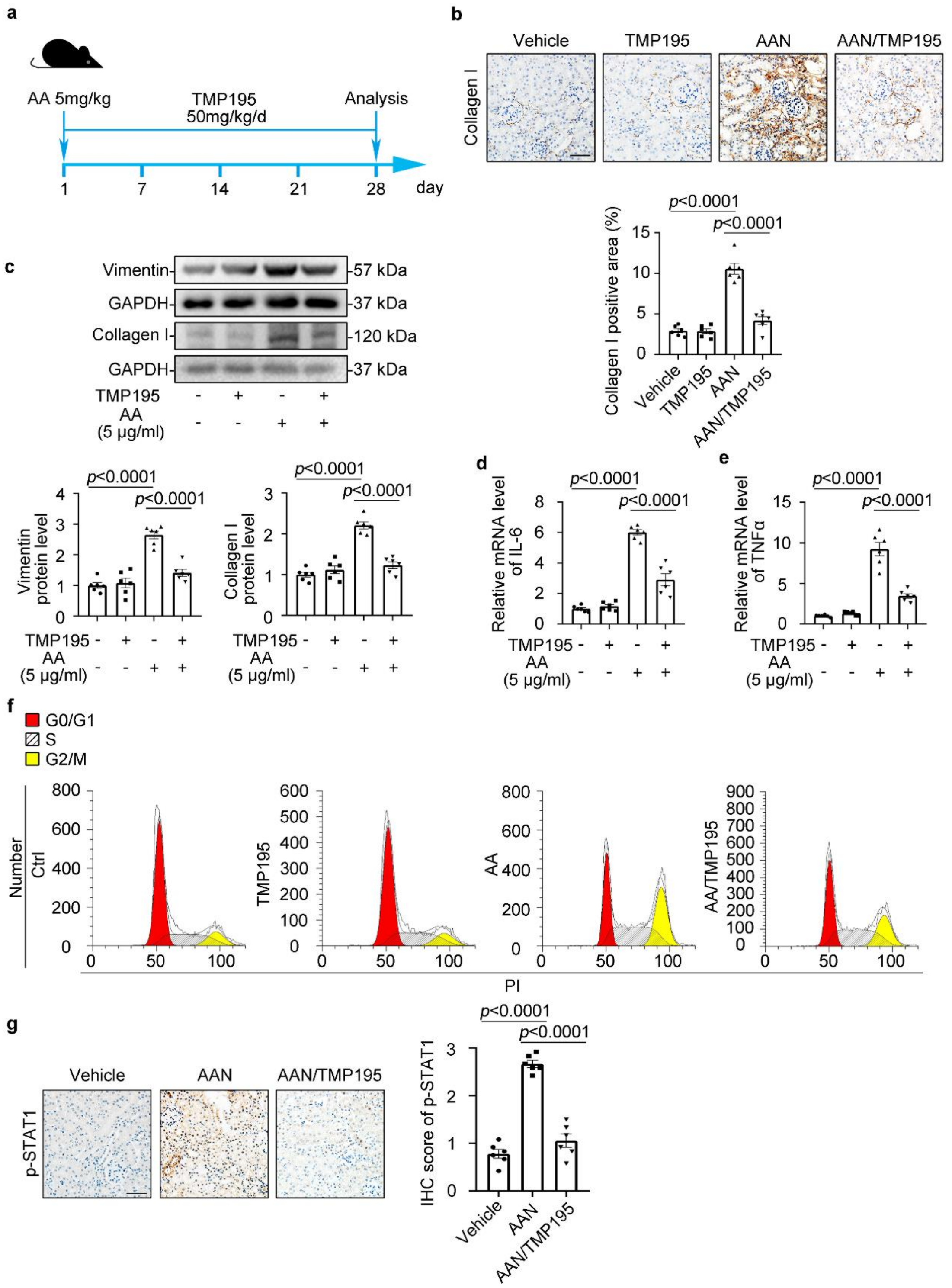

Supplementary Figure S11. TMP195 attenuated kidney fibrosis and inhibited G2/M phase arrest in TECs. a. A schematic diagram showing TMP195 treatment in AAN mice. **b.** Photomicrographs and quantifications showing the expression of collagen I in kidney from different groups of mice. Scale bar: black = 50 μm. (n = 6 mice per group). **c.** Representative Western blot gel documents and summarized data showing the relative protein levels of Vimentin and Collagen I in HK-2 with AA treatments. (n = 6 biologically independent experiments). **d and e.** Relative mRNA

level of *IL-6* and *TNFα* in HK-2 with different treatments. (n = 6 biologically independent experiments). **f**. Cell cycle analysis by flow cytometry for HK-2 in different groups. **g.** Photomicrographs and quantifications showing the protein levels of p-STAT1 in kidney from different groups of mice. Scale bar: black = 50 µm. (n = 6 mice per group). Data are expressed as mean ± SEM (b, c, d, e and g). Two-way ANOVA followed by Tukey's post-test (b-e and g). Source data are provided as a Source Data file.

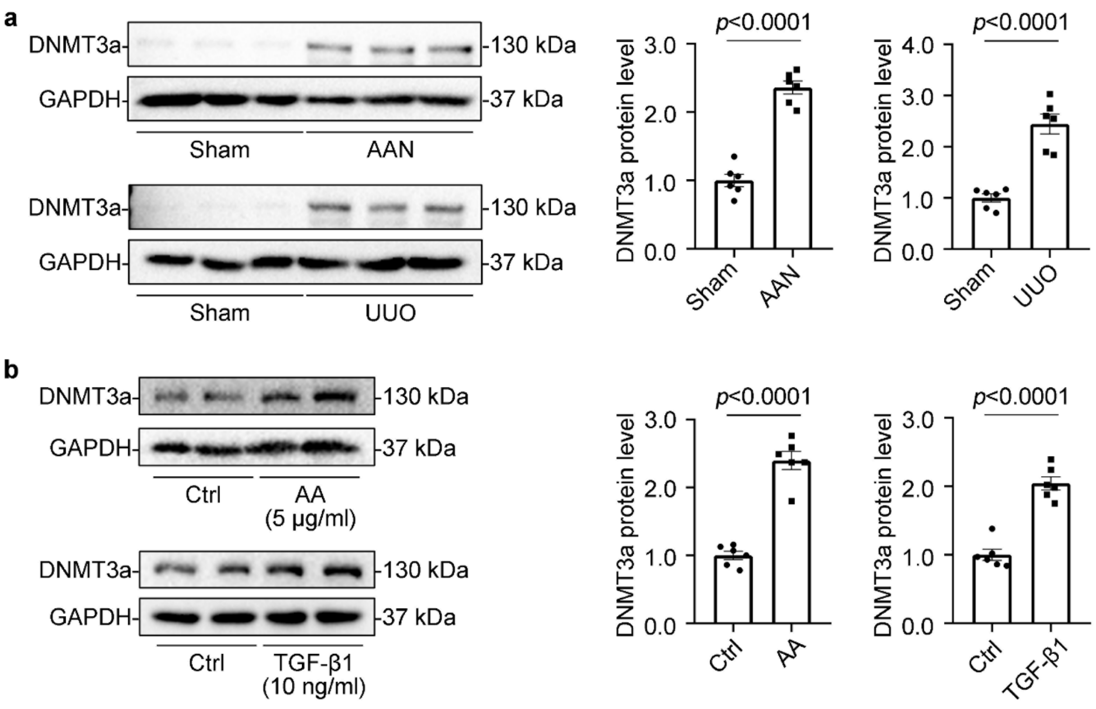

Supplementary Figure S12. DNMT3a was increased in fibrotic kidneys and HK-2 with AA or TGF-β1 treatment. a. Representative Western blot gel documents and summarized data showing the relative protein levels of DNMT3a in the cortex of kidney from AAN or UUO mice. (n = 6 mice per group). **b.** Representative Western blot gel documents and summarized data showing the relative protein levels of DNMT3a in HK-2 with different treatments. (n = 6 biologically independent experiments). Data are expressed as mean ± SEM (a and b). Two-tailed Student's unpaired t test analysis (a and b). Source data are provided as a Source Data file.
