# Supplementary material for: HDAC9-mediated epithelial cell cycle arrest in G2/M contributes to kidney fibrosis in male mice
Source: Nat Commun. 2023 May 25;14:3007. doi: 10.1038/s41467-023-38771-4 (PMC10212923; doi:10.1038/s41467-023-38771-4)
Supplement: Supplementary file 3 — Reporting Summary [file 41467_2023_38771_MOESM3_ESM.pdf]

nature portfolio

Corresponding author(s): DBPR NCOMMS-22-47548B, Fan Yi

Last updated by author(s): Apr 7, 2023

Reporting Summary

Nature Portfolio wishes to improve the reproducibility of the work that we publish. This form provides structure for consistency and transparency in reporting. For further information on Nature Portfolio policies, see our Editorial Policies and the Editorial Policy Checklist.

Statistics

For all statistical analyses, confirm that the following items are present in the figure legend, table legend, main text, or Methods section.

n/a	Confirmed	
☐	☒	The exact sample size (*n*) for each experimental group/condition, given as a discrete number and unit of measurement
☐	☒	A statement on whether measurements were taken from distinct samples or whether the same sample was measured repeatedly
☐	☒	The statistical test(s) used AND whether they are one- or two-sided<br>*Only common tests should be described solely by name; describe more complex techniques in the Methods section.*
☒	☐	A description of all covariates tested
☐	☒	A description of any assumptions or corrections, such as tests of normality and adjustment for multiple comparisons
☐	☒	A full description of the statistical parameters including central tendency (e.g. means) or other basic estimates (e.g. regression coefficient) AND variation (e.g. standard deviation) or associated estimates of uncertainty (e.g. confidence intervals)
☐	☒	For null hypothesis testing, the test statistic (e.g. *F*, *t*, *r*) with confidence intervals, effect sizes, degrees of freedom and *P* value noted<br>*Give P values as exact values whenever suitable.*
☒	☐	For Bayesian analysis, information on the choice of priors and Markov chain Monte Carlo settings
☒	☐	For hierarchical and complex designs, identification of the appropriate level for tests and full reporting of outcomes
☐	☒	Estimates of effect sizes (e.g. Cohen's *d*, Pearson's *r*), indicating how they were calculated

Our web collection on statistics for biologists contains articles on many of the points above.

Software and code

Policy information about availability of computer code

Data collection	Bio-Rad CFX Manager 2.1 (Bio-Rad, California, USA), CytExpert 2.4 (Beckman Coulter, California, USA), cellSens (Olympus, Tokyo, Japan), ZEN 2.3 (ZEISS, Oberkochen, Germany), BPTerm10AU BP-2010 (Softron Tokyo, Japan), Qubit® 2.0 Fluorometer (Thermo Fisher Scientific, Waltham, USA), Agilent 2100 bioanalyzer (Agilent Technologies, California, USA).
Data analysis	ModFit LT5.0 software (Verity Software House, Topsham, ME, USA), Image J 1.45 software (National Institutes of Health, Bethesda, USA), Hisat2 (version:2.0.4), Stringtie (version:1.3.0), GraphPad Prism 8.0 (GraphPad Software, California, USA).

For manuscripts utilizing custom algorithms or software that are central to the research but not yet described in published literature, software must be made available to editors and reviewers. We strongly encourage code deposition in a community repository (e.g. GitHub). See the Nature Portfolio guidelines for submitting code & software for further information.

Data

Policy information about availability of data

All manuscripts must include a data availability statement. This statement should provide the following information, where applicable:

- Accession codes, unique identifiers, or web links for publicly available datasets
- A description of any restrictions on data availability
- For clinical datasets or third party data, please ensure that the statement adheres to our policy

The authors declare that all data supporting the findings of this study are available within the article and its Supplementary information files. RNA-sequencing data

sets have been deposited to Gene Expression Omnibus under accession code GSE217176 (https://www.ncbi.nlm.nih.gov/geo/query/acc.cgi?acc=GSE217176). GRCm38.p4 reference genome in this study is available at the European Nucleotide Archive (https://www.ebi.ac.uk/ena/browser/view/GCA_000001635.6). Source data are provided with this paper.

Human research participants

Policy information about studies involving human research participants and Sex and Gender in Research.

Reporting on sex and gender	We collected the human renal biopsy samples from patients with chronic kidney disease (Supplementary Table S1), including focal segmental glomerulosclerosis (n = 9, 7 males and 2 females), IgA nephropathy (n = 5, 3 males and 2 females) and diabetic nephropathy (n = 3, 3males). No sex or gender analysis was performed due to low sample size.
Population characteristics	Clinical characteristics in the normal human control subjects or subjects with chronic kidney disease are provided in Supplementary Table S1.
Recruitment	The samples of renal biopsies were obtained from Department of Nephrology, Qilu Hospital of Shandong University, Department of Pathology, Shandong University School of Basic Medical Sciences and Department of Nephrology, the First Affiliated Hospital of Zhengzhou University. The chronic kidney disease patients did not start dialysis therapy at the time of kidney biopsy. Normal control samples were obtained from healthy kidney poles of individuals who underwent tumor nephrectomies or renal cystectomy without other kidney diseases.
Ethics oversight	The investigations were conducted in accordance with the principles of the Declaration of Helsinki and were approved by the Research Ethics Committee of Shandong University (Document No. ECSBMSSDU2018-1-051). The written, informed consent to participate was obtained from all study participants (or their parents/legal guardians). All the human study participants did not receive compensation.

Note that full information on the approval of the study protocol must also be provided in the manuscript.

Field-specific reporting

Please select the one below that is the best fit for your research. If you are not sure, read the appropriate sections before making your selection.

☒ Life sciences ☐ Behavioural & social sciences ☐ Ecological, evolutionary & environmental sciences

For a reference copy of the document with all sections, see nature.com/documents/nr-reporting-summary-flat.pdf

Life sciences study design

All studies must disclose on these points even when the disclosure is negative.

Sample size	Samples sizes were determined based on pilot studies , or based on previous experience with similar experiments (PMID: 36316334, Nat Commun. 2022 Oct 31;13(1):6502). Sample sizes for each experiment are described in figure legends. For in vitro assays, n≥3. For animal experiments, usually n≥5 mice were used.
Data exclusions	No data was excluded from the manuscript.
Replication	Experiments were repeated with the same conditions and obtained similar results. The number of repeats were indicated in figure legends
Randomization	Mice were randomly allocated among groups. For in vitro studies, the experiment was carried out strictly in accordance with the single variable principle. Treatment groups were divided randomly and equally.
Blinding	All the Investigators were blinded to the allocation of different groups during data collection and analysis.

Reporting for specific materials, systems and methods

We require information from authors about some types of materials, experimental systems and methods used in many studies. Here, indicate whether each material, system or method listed is relevant to your study. If you are not sure if a list item applies to your research, read the appropriate section before selecting a response.

Materials & experimental systems

n/a	Involved in the study
☐	☒ Antibodies
☐	☒ Eukaryotic cell lines
☒	☐ Palaeontology and archaeology
☐	☒ Animals and other organisms
☒	☐ Clinical data
☒	☐ Dual use research of concern

Methods

n/a	Involved in the study
☒	☐ ChIP-seq
☐	☒ Flow cytometry
☒	☐ MRI-based neuroimaging

Antibodies

Antibodies used

1. Anti-human/mouse HDAC9 (ORIGENE, Cat#TA324378), dilution: 1:500 for Western blot; dilution: 1:50 for IHC; dilution: 1:50 for IF
2. Anti-human Fibronectin (ProteinTech Group, Cat#15613-1-AP), dilution: 1:1000 for Western blot
3. Anti-human/mouse/Rat Vimentin (Abcam, Cat# ab92547), dilution: 1:100 for IHC; dilution: 1:100 for IF
4. Anti-human/mouse/Rat Vimentin (Cell Signaling Technology, Cat# 5741), dilution: 1:1000 for Western blot
5. Anti-human/mouse/Rat Alpha-smooth muscle (Abcam, Cat# ab124964), dilution: 1:2000 for Western blot; dilution: 1:100 for IHC; dilution: 1:100 for IF
6. Anti-mouse/Rat PCNA (ProteinTech Group, Cat#10205-2-AP), dilution: 1:1000 for Western blot; dilution: 1:50 for IHC; dilution: 1:100 for IF
7. Anti-human/Rat Collagen I (Affinity, Cat# AF7001), dilution: 1:1000 for Western blot
8. Anti-mouse/Rat Collagen I (Cell Signaling Technology, Cat# 72026), dilution: 1:50 for IHC; dilution: 1:50 for IF
9. Anti-mouse Collagen IV (Abcam, Cat# ab236640), dilution: 1:50 for IHC
10. Anti-human Cyclin B1 (Cell Signaling Technology, Cat# 4135), dilution: 1:50 for IHC; dilution: 1:50 for IF
11. Anti-human/mouse Cyclin B1 (ProteinTech Group, Cat# 55004-1-AP), dilution: 1:1000 for Western blot
12. Anti-human/mouse Cyclin D1 (Abcam, Cat# ab16663), dilution: 1:1000 for Western blot
13. Anti-mouse Ki-67 (Abcam, Cat# ab15580), dilution: 1:50 for IF
14. Anti-human/mouse Histone H3 (phospho S10) (Abcam, Cat# ab14955), dilution: 1:50 for IF
15. Anti-mouse PDGF Receptor β (Cell Signaling Technology, Cat# 3169), dilution: 1:50 for IF
16. Anti-mouse TGF beta 1 (Abcam, Cat# ab215715), dilution: 1:50 for IHC
17. Anti-human/mouse TGF beta 1 (Abcam, Cat# ab179695), dilution: 1:2000 for Western blot
18. Anti-human/mouse STAT1 (phospho S727) (Abcam, Cat# ab109461), dilution: 1:1000 for Western blot; dilution: 1:50 for IHC
19. Anti-human Acetylated-Lysine Antibody (Cell Signaling Technology, Cat# 9441), dilution: 1:1000 for Western blot
20. Anti-human STAT1 (Cell Signaling Technology, Cat# 9176), 10μg for IP assay;
dilution: 1:1000 for Western blot
21. Anti-human/mouse STAT1 (Abcam, Cat# ab234400), dilution: 1:1000 for Western blot
22. Anti- mouse STAT1 (Abcam, Cat# ab155933), 10μg for IP assay; dilution: 1:1000 for Western blot
23. Anti-human/mouse p21 (Abcam, Cat# ab109199), dilution: 1:1000 for Western blot; dilution: 1:50 for IF
24. Anti-human STAT1 (phospho S727) (Cell Signaling Technology, Cat# 9177), dilution: 1:50 for IF
25. Anti-human/mouse HDAC3 (ABclonal, Cat# A2139), dilution: 1:1000 for Western blot
26. Anti-human/mouse HDAC7 (Abcam, Cat# ab166911), dilution: 1:1000 for Western blot
27. Anti-human/mouse HDAC8 (ABclonal, Cat# A5829), dilution: 1:1000 for Western blot
28. Anti-mouse F4/80 (Cell Signaling Technology, Cat# 70076), dilution: 1:50 for IHC
29. Anti-human/mouse DNMT3a (Cell Signaling Technology, Cat# 3598), dilution: 1:1000 for Western blot
30. Anti-human STAT2 (ABclonal, Cat# A3588), dilution: 1:1000 for Western blot
31. Anti-human STAT3 (ABclonal, Cat# A1192), dilution: 1:1000 for Western blot
32. Anti-human STAT4 (ABclonal, Cat# A4523), dilution: 1:1000 for Western blot
33. Anti-human STAT5 (ABclonal, Cat# A5029), dilution: 1:1000 for Western blot
34. Anti-human STAT6 (ABclonal, Cat# A19120), dilution: 1:1000 for Western blot
35. Anti-human STAT2 (phospho Y690) (Abcam, Cat# ab191601), dilution: 1:1000 for Western blot
36. Anti-human STAT3 (phospho Tyr705) (Cell Signaling Technology, Cat# 9145), dilution: 1:1000 for Western blot
37. Anti-human STAT4 (phospho Y690) (Santa Cruz Biotechnolog, Cat# sc-28296), dilution: 1:500 for Western blot
38. Anti-human STAT5 (phospho Tyr694) (Cell Signaling Technology, Cat# 4322), dilution: 1:500 for Western blot
39. Anti-human STAT6 (phospho Y641) (ABclonal, Cat# AP0456), dilution: 1:500 for Western blot
40. Anti- mouse Alpha-smooth muscle (ProteinTech Group, Cat# 67735-1-lg), dilution: 1:100 for IF
41. Anti-human/mouse/Rat GAPDH (Abways Technology, Cat# AB0037), dilution: 1:10000 for Western blot
42. Anti-human/mouse β-actin (Abways Technology, Cat# AB0035), dilution: 1:10000 for Western blot
43. Anti-mouse AQP1 (Abcam, Cat# ab9566), dilution: 1:100 for IF
44. Anti-mouse THP (Santa Cruz Biotechnolog, Cat# sc-271022), dilution: 1:100 for IF
45. Anti-mouse Lotus Tetragonolobus Lectin (LTL) (Vector Labs, Cat# B-1325), dilution: 1:200 for IF
46. Anti-mouse Dolichos Biflorus Agglutinin (DBA) (Vector Labs, Cat# B-1035), dilution: 1:200 for IF
47. Anti-Mouse (G3A1) mAb IgG1 Isotype Control (Cell Signaling Technology, Cat# 5415), 10μg for IP assay
48. Mouse Anti-rabbit IgG (Conformation Specific) (L27A9) mAb (HRP Conjugate) (Cell Signaling Technology, Cat# 5127), dilution: 1:2000 for Western blot
49. Goat Anti-Rabbit IgG (H+L) HRP (Abways Technology, Cat# AB0101), dilution: 1:10000 for Western blot
50. Goat Anti-Mouse IgG (H+L) HRP (Abways Technology, Cat# AB0102), dilution: 1:10000 for Western blot
51. Goat Anti-Rabbit IgG H&L (Alexa Fluor® 594) (Abcam, Cat# ab150080), dilution: 1:200 for IF
52. Goat Anti-Mouse IgG H&L (Alexa Fluor® 594) (Abcam, Cat# ab150116), dilution: 1:200 for IF
53. Goat Anti-Rabbit IgG H&L (Alexa Fluor® 488) (Abcam, Cat# ab150077), dilution: 1:200 for IF
54. Alexa Fluor® 488 Streptavidin (YEASEN, Cat# 35103ES60), dilution: 1:200 for IF

Validation

The antibodies used in this study are commercially available and validated by the manufacture. Based on the catalog number, the information of these antibody, including validation statements, relevant citations and antibody profiles in online databases, is available in the manufacture's website.
1. Anti-human/mouse HDAC9 (ORIGENE, Cat#TA324378), https://www.origene.com.cn/catalog/antibodies/primary-antibodies/ta324378/hdac9-rabbit-polyclonal-antibody
2. Anti-human Fibronectin (ProteinTech Group, Cat#15613-1-AP), https://www.ptgcn.com/products/FN1-Antibody-15613-1-AP.htm#tested-applications
3. Anti-human/mouse/Rat Vimentin (Abcam, Cat# ab92547), https://www.abcam.cn/products/primary-antibodies/vimentin-antibody-epr3776-cytoskeleton-marker-ab92547.html
4. Anti-human/mouse/Rat Vimentin (Cell Signaling Technology, Cat# 5741), https://www.cellsignal.cn/products/primary-antibodies/vimentin-d21h3-xp-rabbit-mab/5741?site-search-type=Products&N=4294956287&Ntt=5741&fromPage=plp&_requestid=2172416
5. Anti-human/mouse/Rat Alpha-smooth muscle (Abcam, Cat# ab124964), https://www.abcam.cn/products/primary-antibodies/alpha-smooth-muscle-actin-antibody-epr5368-ab124964.html
6. Anti-mouse/Rat PCNA (ProteinTech Group, Cat#10205-2-AP), https://www.ptgcn.com/products/PCNA-Antibody-10205-2-AP.htm
7. Anti-human/Rat Collagen I (Affinity, Cat# AF7001), https://www.affbiotech.cn/goods-2057-AF7001-Collagen_I_Antibody.html
8. Anti-mouse/Rat Collagen I (Cell Signaling Technology, Cat# 72026), https://www.cellsignal.cn/products/primary-antibodies/col1a1-e8f4l-xp-rabbit-mab/72026?site-search-type=Products&N=4294956287&Ntt=72026&fromPage=plp&_requestid=2174141
9. Anti-mouse Collagen IV (Abcam, Cat# ab236640), https://www.abcam.cn/products/primary-antibodies/collagen-iv-antibody-epr22911-127-ab236640.html
10. Anti-human Cyclin B1 (Cell Signaling Technology, Cat# 4135), https://www.cellsignal.cn/products/primary-antibodies/cyclin-b1-v152-mouse-mab/4135?site-search-type=Products&N=4294956287&Ntt=4135&fromPage=plp&_requestid=2174274
11. Anti-human/mouse Cyclin B1 (ProteinTech Group, Cat# 55004-1-AP), https://www.ptgcn.com/products/CCNB1-Antibody-55004-1-AP.htm
12. Anti-human/mouse Cyclin D1 (Abcam, Cat# ab16663), https://www.abcam.cn/products/primary-antibodies/cyclin-d1-antibody-sp4-ab16663.html
13. Anti-mouse Ki-67 (Abcam, Cat# ab15580), https://www.abcam.cn/products/primary-antibodies/ki67-antibody-ab15580.html
14. Anti-human/mouse Histone H3 (phospho S10) (Abcam, Cat# ab14955), https://www.abcam.cn/products/primary-antibodies/histone-h3-phospho-s10-antibody-mabcam-14955-ab14955.html
15. Anti-mouse PDGF Receptor β (Cell Signaling Technology, Cat# 3169), https://www.cellsignal.cn/products/primary-antibodies/pdgf-receptor-b-28e1-rabbit-mab/3169?site-search-type=Products&N=4294956287&Ntt=3169&fromPage=plp&_requestid=2174781
16. Anti-mouse TGF beta 1 (Abcam, Cat# ab215715), https://www.abcam.cn/products/primary-antibodies/tgf-beta-1-antibody-epr21143-ab215715.html
17. Anti-human/mouse TGF beta 1 (Abcam, Cat# ab179695), https://www.abcam.cn/products/primary-antibodies/tgf-beta-1-antibody-epr18163-ab179695.html
18. Anti-human/mouse STAT1 (phospho S727) (Abcam, Cat# ab109461), https://www.abcam.cn/products/primary-antibodies/stat1-phospho-s727-antibody-epr3146-ab109461.html
19. Anti-human Acetylated-Lysine Antibody (Cell Signaling Technology, Cat# 9441), https://www.cellsignal.cn/products/primary-antibodies/acetylated-lysine-antibody/9441?site-search-type=Products&N=4294956287&Ntt=9441&fromPage=plp&_requestid=2175068
20. Anti-human STAT1 (Cell Signaling Technology, Cat# 9176), https://www.cellsignal.cn/products/primary-antibodies/stat1-9h2-mouse-mab/9176?site-search-type=Products&N=4294956287&Ntt=9176&fromPage=plp&_requestid=2175132
21. Anti-human/mouse STAT1 (Abcam, Cat# ab234400), https://www.abcam.cn/products/primary-antibodies/stat1-antibody-epr21057-141-chip-grade-ab234400.html
22. Anti- mouse STAT1 (Abcam, Cat# ab155933), https://www.abcam.cn/products/primary-antibodies/stat1-antibody-15h3-ab155933.html
23. Anti-human/mouse p21 (Abcam, Cat# ab109199), https://www.abcam.cn/products/primary-antibodies/p21-antibody-epr3993-ab109199.html
24. Anti-human STAT1 (phospho S727) (Cell Signaling Technology, Cat# 9177), https://www.cellsignal.cn/products/primary-antibodies/phospho-stat1-ser727-antibody/9177?site-search-type=Products&N=4294956287&Ntt=9177&fromPage=plp&_requestid=2175415
25. Anti-human/mouse HDAC3 (ABclonal, Cat# A2139), https://abclonal.com.cn/catalog/A2139
26. Anti-human/mouse HDAC7 (Abcam, Cat# ab166911), https://www.abcam.cn/products/primary-antibodies/hdac7-antibody-epr10922-ab166911.html
27. Anti-human/mouse HDAC8 (ABclonal, Cat# A5829), https://abclonal.com.cn/catalog/A5829
28. Anti-mouse F4/80 (Cell Signaling Technology, Cat# 70076), https://www.cellsignal.cn/products/primary-antibodies/f4-80-d2s9r-xp-rabbit-mab/70076?site-search-type=Products&N=4294956287&Ntt=70076&fromPage=plp&_requestid=2175864
29. Anti-human/mouse DNMT3a (Cell Signaling Technology, Cat# 3598), https://www.cellsignal.cn/products/primary-antibodies/dnmt3a-d23g1-rabbit-mab/3598?site-search-type=Products&N=4294956287&Ntt=3598&fromPage=plp&_requestid=2176266
30. Anti-human STAT2 (ABclonal, Cat# A3588), https://abclonal.com.cn/catalog/A3588
31. Anti-human STAT3 (ABclonal, Cat# A1192), https://abclonal.com.cn/catalog/A1192
32. Anti-human STAT4 (ABclonal, Cat# A4523), https://abclonal.com.cn/catalog/A4523
33. Anti-human STAT5 (ABclonal, Cat# A5029), https://abclonal.com.cn/catalog/A5029
34. Anti-human STAT6 (ABclonal, Cat# A19120), https://abclonal.com.cn/catalog/A19120
35. Anti-human STAT2 (phospho Y690) (Abcam, Cat# ab191601), https://www.abcam.cn/products/primary-antibodies/stat2-phospho-y690-antibody-epr18549-42-chip-grade-ab191601.html
36. Anti-human STAT3 (phospho Tyr705) (Cell Signaling Technology, Cat# 9145), https://www.cellsignal.cn/products/primary-antibodies/phospho-stat3-tyr705-d3a7-xp-rabbit-mab/9145?site-search-type=Products&N=4294956287&Ntt=9145&fromPage=plp&_requestid=2176825
37. Anti-human STAT4 (phospho Y690) (Santa Cruz Biotechnolog, Cat# sc-28296), https://www.scbt.com/p/p-stat4-antibody-e-2?requestFrom=search
38. Anti-human STAT5 (phospho Tyr694) (Cell Signaling Technology, Cat# 4322), https://www.cellsignal.cn/products/primary-antibodies/phospho-stat5-tyr694-d47e7-xp-rabbit-mab/4322?site-search-type=Products&N=4294956287&Ntt=4322&fromPage=plp&_requestid=2177081
39. Anti-human STAT6 (phospho Y641) (ABclonal, Cat# AP0456), https://abclonal.com.cn/catalog/AP0456
40. Anti- mouse Alpha-smooth muscle (ProteinTech Group, Cat# 67735-1-lg), https://www.ptgcn.com/products/smooth-muscle-

actin-specific-Antibody-67735-1-Ig.htm
41. Anti-human/mouse/Rat GAPDH (Abways Technology, Cat# AB0037), http://www.abways.cn/showproduct.asp?cid=AB0037
42. Anti-human/mouse β-actin (Abways Technology, Cat# AB0035), http://www.abways.cn/showproduct.asp?cid=AB0035
43. Anti-mouse AQP1 (Abcam, Cat# ab9566), https://www.abcam.cn/products/primary-antibodies/aquaporin-1-antibody-122-ab9566.html
44. Anti-mouse THP (Santa Cruz Biotechnolog, Cat# sc-271022), https://www.scbt.com/p/thp-antibody-b-2?requestFrom=search
45. Anti-mouse Lotus Tetragonolobus Lectin (LTL) (Vector Labs, Cat# B-1325), https://vectorlabs.com/products/products/glycobiology/biotinylated-lotus-tetragonolobus-lectin-ltl
46. Anti-mouse Dolichos Biflorus Agglutinin (DBA) (Vector Labs, Cat# B-1035), https://vectorlabs.com/products/products/glycobiology/biotinylated-dolichos-biflorus-agglutinin
47. Anti-Mouse (G3A1) mAb IgG1 Isotype Control (Cell Signaling Technology, Cat# 5415), https://www.cellsignal.cn/products/primary-antibodies/mouse-g3a1-mab-igg1-isotype-control/5415?site-search-type=Products&N=4294956287&Ntt=5415&fromPage=plp&_requestid=2181618
48. Mouse Anti-rabbit IgG (Conformation Specific) (L27A9) mAb (HRP Conjugate) (Cell Signaling Technology, Cat# 5127), https://www.cellsignal.cn/products/secondary-antibodies/mouse-anti-rabbit-igg-conformation-specific-l27a9-mab-hrp-conjugate/5127?site-search-type=Products&N=4294956287&Ntt=5127&fromPage=plp&_requestid=2181675
49. Goat Anti-Rabbit IgG (H+L) HRP (Abways Technology, Cat# AB0101), http://www.abways.cn/showproduct.asp?cid=AB0101
50. Goat Anti-Mouse IgG (H+L) HRP (Abways Technology, Cat# AB0102), http://www.abways.cn/showproduct.asp?cid=AB0102
51. Goat Anti-Rabbit IgG H&L (Alexa Fluor® 594) (Abcam, Cat# ab150080), https://www.abcam.cn/products/secondary-antibodies/goat-rabbit-igg-hl-alexa-fluor-594-ab150080.html
52. Goat Anti-Mouse IgG H&L (Alexa Fluor® 594) (Abcam, Cat# ab150116), https://www.abcam.cn/products/secondary-antibodies/goat-mouse-igg-hl-alexa-fluor-594-ab150116.html
53. Goat Anti-Rabbit IgG H&L (Alexa Fluor® 488) (Abcam, Cat# ab150077), https://www.abcam.cn/products/secondary-antibodies/goat-rabbit-igg-hl-alexa-fluor-488-ab150077.html
54. Alexa Fluor® 488 Streptavidin (YEASEN, Cat# 35103ES60), https://www.yeasen.com/products/detail/268

Eukaryotic cell lines

Policy information about cell lines and Sex and Gender in Research

Cell line source(s)

Human tubule epithelial cells (HK-2) and normal rat kidney fibroblast cells (NRK-49F) were pruchased from American Type Culture Collection (ATCC).

Authentication

Human tubule epithelial cells (HK-2) and normal rat kidney fibroblast cells (NRK-49F) were authenticated by American Type Culture Collection (ATCC). Cell lines were authenticated by STR profiling.

Mycoplasma contamination

We tested and confirmed all used cell lines are mycoplasma negative.

Commonly misidentified lines (See ICLAC register)

No commonly misidentified lines were used in this study.

Animals and other research organisms

Policy information about studies involving animals; ARRIVE guidelines recommended for reporting animal research, and Sex and Gender in Research

Laboratory animals

Global HDAC9 knockout (HDAC9 +/-) mice (C57BL/6JSmoc-HDAC9em1Smoc, Shanghai Model Organisms Center, Inc., Shanghai, China) were purchased from Shanghai Model Organisms. Floxed HDAC9 mice(C57BL/6JSmoc-HDAC9em1(flox)Smoc, Shanghai Model Organisms Center, Inc., Shanghai, China) were hybridized with transgenic mice expressing Cre-recombinase under the cadherin 16 promoter (B6.Cg–Tg(Cdh16-cre)91Igr/J, Jackson Laboratory) to generated tubule-specific HDAC9 knockout mice (Cdh16-Cre+/HDAC9fl/fl; Cre+/HDAC9fl/fl). Age-matched mice without Cre (Cdh16-Cre-/ HDAC9fl/fl; Cre-/HDAC9fl/fl) were used as controls.

All experimental protocols for animal studies were conducted in accordance with the National Institutes of Health Guide for the Care and Use of Laboratory Animals and were approved by the Institutional Animal Care and Use Committee of School of Basic Medical Sciences, Shandong University (Document No. ECSBMSSDU2018-2-091). All mice (3-5 mice per cage) were housed under SPF conditions (12 h light/dark cycle, 24 °C and 40–60% humidity) with ad libitum access to water and standard laboratory chow diet (Beijing KEAOXIELI feed company, Beijing, China). Water and cages were autoclaved. Cages with standard corncob bedding were changed three times a week. For all of the in vivo experiments, littermate control mice were used. All of our experimental animals were kept under barrier conditions under constant veterinary supervision and did not display signs of distress or pathological changes that warranted veterinary intervention. Different groups were allocated in a randomized manner and investigators were blinded to the allocation of different groups when doing surgeries and doing outcome evaluations. The number of the mice used for the experiments is indicated for each experiment in the figure legends. An established mouse model of renal IRI was performed. Briefly, Male C57BL/6 mice, aged 8 weeks (24-28g), were anesthetized with an intraperitoneal injection of pentobarbital sodium (30 mg/kg body weight) and maintained on a heat pad during surgery. A midline abdominal incision was made and bilateral renal pedicles were clipped for 32 min at 37.5 °C (bilateral IRI, BIRI) or only the left kidney for 35 min at 37 °C (unilateral IRI, UIRI). Sham operations were performed with exposure of both kidneys but without induction of ischemia. After surgery, the mice were maintained under SPF conditions (12 h light/dark cycle, 24 °C and 40–60% humidity) with ad libitum access to water and standard laboratory chow diet. The mice were euthanized and the kidney tissue samples were harvested for histopathological analysis after 4 weeks. The UUO model (Male C57BL/6 mice, aged 8 weeks, 24-28g) was generated by ligation of the left ureter. After surgery, the mice were maintained under SPF conditions (12 h light/dark cycle, 24 °C and 40–60% humidity) with ad libitum access to water and standard laboratory chow diet. After 7 days of ureteral obstruction, the mice were euthanized and the kidney tissue samples were harvested for histopathological analysis. For the aristolochic acid nephropathy, male C57BL/6 mice, aged 8 weeks (24-28g), were used. The animal model was induced by a one-time intraperitoneal injection of aristolochic acid (5 mg/kg body weight, A5512, Sigma-

	Aldrich) in PBS. The normal control mice were administered the same amount of PBS. After administration, the mice were maintained under SPF conditions (12 h light/dark cycle, 24 °C and 40–60% humidity) with ad libitum access to water and standard laboratory chow diet. The mice were euthanized and the kidney tissue samples were harvested for histopathological analysis after 28 days.
Wild animals	No wild animals were used in this study.
Reporting on sex	It has been reported that male gender is associated with a more rapid rate of progression of nondiabetic chronic renal disease (PMID: 10665939). Due to the difference in susceptibility to aristolochic acid nephropathy (PMID: 27763560) and unilateral ureter obstruction (PMID: 22143161) between male and female mice, we firstly selected male mice in the present studies.
Field-collected samples	No field-collected animals were used in the study.
Ethics oversight	All experimental protocols for animal studies were conducted in accordance with the National Institutes of Health Guide for the Care and Use of Laboratory Animals and were approved by the Institutional Animal Care and Use Committee of School of Basic Medical Sciences, Shandong University (Document No. ECSBMSSDU2018-2-091).

Note that full information on the approval of the study protocol must also be provided in the manuscript.

Flow Cytometry

Plots

Confirm that:

☒ The axis labels state the marker and fluorochrome used (e.g. CD4-FITC).

☒ The axis scales are clearly visible. Include numbers along axes only for bottom left plot of group (a 'group' is an analysis of identical markers).

☒ All plots are contour plots with outliers or pseudocolor plots.

☒ A numerical value for number of cells or percentage (with statistics) is provided.

Methodology

Sample preparation	HK-2 cells were harvested and fixed at 4°C overnight with 70% ethanol. According to the manufacturer’s protocol (KeyGEN BioTECH, Jiangsu, China), the fixed cells were incubated with RNase A and PI for 30-60 min at room temperature (25°C) in the dark after washing and analyzed by flow cytometry (CytoFLEX, Beckman Coulter, CytExpert 2.4 version). ModFit LT 5.0 software (Verity Software House, Topsham, ME, USA) was used to analyze cell cycle distribution.
Instrument	Flow cytometry (CytoFLEX, Beckman Coulter)
Software	ModFit LT 5.0 software (Verity Software House, Topsham, ME, USA), CytExpert 2.4 (Beckman Coulter, California,USA)
Cell population abundance	Cell population abundance is determined by ModFit LT 5.0 software (Verity Software House, Topsham, ME, USA)
Gating strategy	The FACS assay used in this study is an established protocol for cell cycle ananlysis.

☒ Tick this box to confirm that a figure exemplifying the gating strategy is provided in the Supplementary Information.
